# How best to measure tear film formation: an opinion

**DOI:** 10.3389/fphys.2026.1833744

**Published:** 2026-06-16

**Authors:** Timon Ax, Slade O. Jensen, Thomas J. Millar

**Affiliations:** 1 School of Medicine, Western Sydney University, Campbelltown, NSW, Australia; 2 Antimicrobial Resistance and Mobile Elements Group, Ingham Institute of Applied Medical Research, Liverpool, NSW, Australia; 3 Beyond 700 Pty Ltd., Sydney, NSW, Australia

**Keywords:** lipid layer, tear film, tear film dynamics, tear film formation, tear film imaging, tear film parameters, tear film particles, tear film physiology

## Introduction

Healthy eyes are covered by a tear film that is stable and renewed with each blink. A functional tear film has a surface lipid layer blanketing a complex aqueous layer that contains various proteins and mucins. Approximately 5%–50% of people suffer from a condition known as dry eye disease, in which the tear film is unstable (Stapleton et al., 2017). Clinically, the gold standard in diagnostic tests is the tear film break-up time that determines how quickly the tear film breaks down after a blink. There has been extensive research into how and why the tear film is unstable in patients with dry eye disease (King-Smith et al., 2018). Some investigators, on the other hand, have examined how quickly the tear film forms upon blinking, typically referred to as tear film build-up time (Nemeth et al., 2002). Such studies aim to provide insight into the physiology of the tear film and why healthy tear films are stable and resilient across different environmental conditions. Knowing when the tear film has fully formed is clinically relevant for biometry and retinal imaging because this is when the optical quality of the eye is optimal (Montés-Micó et al., 2010).

## Considerations when examining tear film formation

Following a blink, tear film formation occurs in two steps (Holly, 1973). First, as the upper lid rises, the cornea is initially covered by the aqueous and mucin layers, followed by a slower upward motion of the lipid layer that thickens the tear film and redistributes the mucoaqueous layer to the upper part of the ocular surface (Brown and Dervichian, 1969). The second and final step of tear film formation can be imaged.

Measuring tear film formation *in vivo* has been inconsistent (**Table 1**) but this is expected because of the many considerations which influence experimental design.

**Table 1:** Summary of studies of tear film formation.

Reference	*n* (healthy subjects); *n* (female); special conditions	Mean age (years)	Blinking conditions	Imaging device; frame rate; resolution; illumination	Time origin, *t*_0_	Analysis	Results: tear film spreading velocity; time to stabilization
Garcia-Marques et al., 2023	84; 52 f; right eyes only	22.4 ± 2.6; range: 18–27	Spontaneous, complete and natural blinks (NBC)	Oculus Keratograph 5M (Wetzlar, Germany); 32 fps; 1,360 × 1,024 pixel; illuminated by 2 white LEDs	Frames after a spontaneous, complete, and natural blink	Particles tracked using MatLab; particles’ speed within 1.75 s post-blink; at least 8 different particles tracked and averaged	1.1 ± 0.4 mm/s (median particle speed); 1.1 ± 0.4 s (time for particle speed to decrease to <1.20 mm/s)
Lai et al., 2023	36; 27 f (DED w/o SS) and 32 (DED + SS); 32 f	61.38 ± 9.91 (DED w/o SS); 60.82 ± 70.91 (DED + SS)	Instructed to blink naturally	Oculus Keratograph 5M (Wetzlar, Germany); 10 fps; ×1.4 magnification	Start of opening cycle of the blink	Momentary movement speed (MMS): Averaged speeds of three reflective particles, then power-law fit operation used to estimate velocity of particles at every moment	MMS (0.01 s)^a^: 60.82 ± 70.91 (DED w/o SS); 16.81 ± 18.82 mm/s (DED + SS) MMS (0.05 s): 9.16 ± 6.69 (DED w/o SS), 3.64 ± 2.90 mm/s (SS) MMS (0.1 s): 4.34 ± 2.49 (DED w/o SS), 2.01 ± 1.39 (DED + SS) MMS (0.5 s): 0.91 ± 0.40 (DED w/o SS), 0.59 ± 0.37 (DED + SS)
Garcia-Marques et al., 2021	81; 46 f; 77 right eyes	43.7 ± 27.0; range: 18–90	Spontaneous, complete and natural blinks (NBC)	Oculus Keratograph 5M (Wetzlar, Germany); 32 fps; 1,360 × 1,024 pixel; illuminated by 2 white LEDs	Frames after a spontaneous, complete, and natural blink	Particles tracked using MatLab; particles’ speed within 1.75 s post-blink; at least 8 different particles tracked and averaged	1.48 ± 0.49 mm/s (mean particle speed), 1.17 ± 0.47 mm/s (median particle speed), 3.37 ± 1.26 mm/s (maximum particle speed), 0.66 ± 0.34 mm/s (minimum particle speed)
Szczesna-Iskander, 2018	19; 9 f; right eyes only, left eyes were patched	29 ± 9; range: 20–57	Natural, spontaneous blinking	Lateral shearing interferometry (LSI); 25 fps; central corneal area ~4 to 5 mm in diameter	After a blink	Measurement of tear film surface quality (TFSQ)	Only decay parameters provided; figures show highest quality of tear film surface at 1–2.5 s
Ring et al., 2012	19; 15 f; mild dry eye, OSDI: 0–15	55.2 ± 16.4	Conditions not specified	USB microscope (PCE-MM200 Microscope; PCE Group, Meschede, Germany) 640 × 480 pixels; 20 fps; diffuse white light source	Onset of opening phase of a blink	Lipid layer stabilization time: mean of five consecutive blinks determined manually as time between *t*_0_ and time when movement of lipid layer stopped. Then, correction of lipid layer stabilization time by the ratio between eyelid distance and iris diameter	Lipid layer stabilization time: 1.11 s (uncorrected), 2.10 s (corrected)
Varikooty et al., 2012	10 silicone hydrogel contact lens wearers; sex not specified	Age not specified	Subjects blinked normally	CCD color video camera (Sony DXC-C33, Sony Corporation) mounted on Zeiss slit-lamp; 30 fps	Not specified	Using the software Fiji, highly reflective tear-film particles through successive frames during one inter-blink interval tracked	Max. ~3.5 mm/s (particle velocity, from their Figure 1), movement stopped approximately ~1 s post-blink
King-Smith et al., 2009	16; 9 f; right eyes only	30 ± 9	Voluntary blink 1 s into measurement, then open eyes for up to 30 s	Doane’s interferometer; 30 fps	Analysis started short time after blink, on average after 0.22 s	Finding maxima of cross-correlation functions between succeeding images; analysis started a short time after the blink, on average after 0.22 s	Velocity of the initial rapid upward movement not given but mostly stabilized ~2 s their (Figure 3); also observed slower steady upward movements and considerable horizontal movements
Yokoi et al., 2008	4; sex not specified; 5 eyes	54.8 ± 4.7	Natural blinking	Video-interferometer (DR-1; Kowa, Tokyo, Japan); 20 fps; low-magnification 7 mm mode	After a blink	Lipid layer clipped along delineated border	No velocity values or time to stabilization values provided
Koh et al., 2006	20; 11 f	30.0 ± 4.4	Forced to blink every 10 s and to keep their eyes open (SBC)	Hartmann–Shack wavefront aberrometer (Topcon, Corp., Tokyo, Japan); 1 Hz/fps	Post blink	*T*_min_ defined as the time at which the root mean square of the ocular total HOAs reached the minimum in each post-blink measurement	*T*_min_: 5.6 ± 2.8 s (stable pattern group), 4.9 ± 1.7 s (small-fluctuation pattern group)
Zhu et al., 2006	10; sex not specified; left eyes only	24.8 (range: 21–30)	Natural blinks “whenever necessary”; breathing at 0.5 Hz guided by electronic metronome while looking naturally at fixation target	High-speed videokeratoscope system based on Medmont E300 videokeratoscope; 50 fps	First available videokeratograph directly after blink that had complete information and good quality ring pattern	Calculation of ocular surface height maps; ocular surface height difference maps were derived by subtracting baseline ocular surface height map from series of sequential maps to reveal and highlight changes in distribution of tear film	Within first 0.5 s, ocular surface height significantly increased at top of topography map and decreased in the inferior region; ocular surface topography became relatively stable 2-3 s post-blink
Erdelyi et al., 2006; same data as Nemeth et al., 2002	15; 12 f	31.5 ± 10.4	After a complete blink, then suppressed blinking conditions	Videokeratoscope (model TMS-1); 4 fps	First corneal image of sequence (first image where eye was not closed) was excluded from statistical analysis	Surface regularity index (SRI): topography of central part of the cornea—approximately 3.5-mm diameter; surface asymmetry index (SAI): measurement of regularity of overall ocular surface	SRI decreased to minimum value (SRI_min_), which occurred at time (*t*_min_) of 7.1 ± 3.9 s after complete blink; SAI_min_ at 5.43 ± 2.7 s
Montes-Mico et al., 2004	15; 2 f; left eyes only	26 ± 1.7; range: 24 to 33	Eyelids kept open during image capture (SBC)	Tomey TMS-2N corneal topographer (Tomey Corp., Nagoya, Japan), 1 Hz/fps	After a blink	Total amounts of higher-order (third to sixth) RMS (root mean square) wavefront aberration of the anterior cornea	Minimum level at 6.1 ± 0.5 s
Goto and Tseng, 2003	11; 4 f; random side	38.7 ± 14.3	Complete blinks	DR1 tear interference camera, 8-mm diameter (×12 magnification); 5.18 fps	First full eye-opening frame	Lipid spread time to reach stable image measure frame by frame by hand	Lipid spread completed in 0.36 ± 0.22 s (i.e., fewer than 2 frames)
Owens and Phillips, 2002	20; 14 f	Range: 18 to 41	Encouraged to blink naturally	Nikon biomicroscope (model FS-3V; Japan) + Sony Handicam video camera CCD-TR3-3E; ×130 magnification; 25 fps	Frame in which cornea was first fully visible after a blink	Particle velocity computed by measuring distance traveled across the screen by individual particles in 0.04 s; when particle disappeared out of view, next particle was tracked	Initial velocity (refers to velocity 40 ms after *t*_0_): 7.34 ± 2.73 mm/s; time to tear stabilization (i.e., zero velocity): 1.05 ± 0.30 s
Nemeth et al., 2002	15; 12 f; right eyes only	31.5 ± 10.4; range: 20–56	Asked to make complete blink and subsequently to keep eyes open and to fixate continuously (SBC)	Videokeratoscope (model TMS-1); 4 fps	After a voluntary complete blink; first corneal image of sequence (first image where eye was not closed) was excluded	Surface regularity index (SRI): topography of the central part of the cornea ~3.5-mm diameter; surface asymmetry index (SAI): measure of the regularity of the overall ocular surface	SRI_min_ at *t*_min_ = 7.1 ± 3.9 s; SAI_min_ at *t*_min=_ 5.4 ± 2.7 s
Benedetto et al., 1984	9; sex not specified	26 with all subjects being less than 35 years, except for one	Allowed to blink involuntarily (NBC)	Fluorophotometer; fluorescence monitored over central 1 mm of cornea	Continuous recording	Tear film fluorescence: intensity of fluorescence of precorneal tear film in arbitrary units	Tear film builds up quickly after eyelids are opened (2 to 3 s); tear film thickened superiorly while thinning inferiorly
Berger and Corrsin, 1974	Not specified	Age not specified	Subjects were requested to blink	Slit-lamp and Bolex camera; 32 fps	Frame where lid was last observed to be fully closed	Particle velocity: Distance of particle from lower lid over time	Early velocities: ~10 mm/s, increasing toward upper lid; motion persists for ~1 s

This table only refers to *in vivo* studies of tear film formation in humans. It reports on a selection of studies deemed most relevant, and included, with few exceptions, normal subjects. The table does not cover mathematical models, animal experiments, or *in vitro* experiments.

NBC, natural blinking conditions; SBC, suppressed blinking conditions; DED, dry eye disease; SS, Sjögren syndrome; HOA, higher-order aberration.

^a^ Modeled values through power-law fitting operation.

*Data from dry eye patients or otherwise non-normal conditions.

Views differ regarding the appropriate start and end points for measurement, and what constitutes a fully formed tear film. Experiments are also constrained by what can be measured. The invisibility and thinness of the tear film have meant that surrogate parameters have been used and not the tear film itself.

The most commonly used surrogate methods for measuring tear film formation involve tracking lipid layer spread and the movement of reflective particles embedded in the lipid layer using interferometry or slit-lamp biomicroscopy. This has allowed the determination of when tear film spread is complete, as indicated by the end of movement of colorful fringes or particles (lipid layer stabilization). Despite differences in experimental details, it takes approximately 1 s for the lipid layer to stabilize following a blink. An outlier study found a lipid spread time of only 0.36 ± 0.22 s (Goto and Tseng, 2003). Few have examined aqueous layer dynamics (Benedetto et al., 1984; Zhu et al., 2006). Other studies measured ocular surface parameters such as surface regularity index, surface asymmetry index, or tear film surface quality to evaluate tear film formation. They determined when these parameters reached their lowest value, i.e., the time when the tear film was optimal. These studies consistently report longer times: 2.5–7 s. A time of 7 s to reach an optimal tear film is unexpected since the average interblink interval is approximately 6 s (Johnston et al., 2013). One publication (King-Smith et al., 2009) reports that the tear film never stabilized, which might be a result of subtle eye and head movements (Szczesna-Iskander and Robert Iskander, 2025). Overall, the techniques have included tear film fluorophotometry, slit-lamp biomicroscopy, high-speed videokeratoscopy, interferometry, and dynamic wavefront sensing (**Table 1**).

Velocity studies of tear film formation commonly use either lipid layer features or particles. King-Smith et al. (2009) identified an initial fast phase and then a slow phase of tear film build-up, whereas Szczesna-Iskander (2018) introduced a multi-phase model of tear film surface kinetics with an initial fast and then a slow tear film build-up followed by a stable phase. Importantly, because the lipid layer spread slows rapidly after a blink, a common time origin (*t*_0_) is required to compare the so-called initial velocities between studies [as recommended by Owens and Phillips (2002)]; this refers to the precise starting point of a measurement. This is not possible when “after a blink” is a frequent descriptor of *t*_0_ (**Table 1**) as the exact end point of a blink is difficult to define due to frequent low-amplitude twitching of the upper eyelid as it approaches nearly fully open position (Kwon et al., 2013).

The position of the particle being measured also requires consideration because more superior particles (more distant from the lower lid) tend to have higher velocities than more inferior particles during and after the upstroke of a blink (Berger and Corrsin, 1974; King-Smith et al., 2009). This means that instantaneous velocity measurements depend on when after a blink they are recorded and where on the ocular surface they are obtained. Moreover, calculating the average velocity using the starting and end point of a particle would be invalid because it does not reflect the substantial changes in velocity over time. It appears that most publications have chosen to track particles near the center of the cornea, presumably because they are more visible and in focus (Owens and Phillips, 2002; Varikooty et al., 2012).

Blinking conditions are another important variable. Normally, there is no awareness of natural blinking. However, once being made aware of blinking or instructed to blink, blinking becomes varied in terms of frequency and force (Palakuru et al., 2007; McMonnies, 2020; Szczesna-Iskander and Llorens Quintana, 2020). While natural blinking conditions (complete and spontaneous) have been used in more recent studies, many studies have employed instructed or actively suppressed blinking (**Table 1**). Surprisingly, blinking conditions have not been described in some publications. This suggests that it was not considered a variable.

## Discussion

There is no gold standard for studying tear film formation. Each publication uses its own unique experimental design, making direct comparisons between studies difficult. Therefore, common baseline factors are needed to allow meaningful comparisons across research. In our view, natural blinking is preferred, and if instructed or suppressed blinking conditions are used, this must be explicitly stated. We also recommend that the turning point of a blink (i.e., the first frame depicting the beginning of eye opening) represents *t*_0_. In terms of measuring velocity, instantaneous velocities relative to *t*_0_ should be recorded. In this regard, having a higher frame rate enhances the precision of turning point identification (Navascues-Cornago et al., 2026). When particles are studied, most publications appear to have chosen to track particles near the center of the cornea (due to their greater visibility and focus). This means there has likely been a bias toward higher velocities. We recommend using particles that are convenient to measure and consistently visible. However, in all cases, the particle position on the eye should be noted relative to the central cornea.

Progress toward understanding the physiology of tear film formation is only possible with a common research foundation that can, in turn, guide future studies in developing standardized and meaningful guidelines for tear film measurements.
